# Genome-Wide Identification and Analysis of TCP Transcription Factors Involved in the Formation of Leafy Head in Chinese Cabbage

**DOI:** 10.3390/ijms19030847

**Published:** 2018-03-14

**Authors:** Yan Liu, Xiaoyu Guan, Shengnan Liu, Meng Yang, Junhui Ren, Meng Guo, Zhihui Huang, Yaowei Zhang

**Affiliations:** 1 College of Horticulture, Northeast Agricultural University, Harbin 150030, China; liuyanworld@126.com (Y.L.); guanxiaoyu0924@126.com (X.G.); 18846035480@163.com (S.L.); yangmeng931105@163.com (M.Y.); 18800437908@163.com (J.R.); guomeng950331@163.com (M.G.); huangzhihui8866@163.com (Z.H.); 2 Key Laboratory of Biology and Genetic Improvement of Horticultural Crops (Northeast Region), Ministry of Agriculture, Harbin 150000, China

**Keywords:** Chinese cabbage, TCP TFs, head formation, leaf curvature

## Abstract

Chinese cabbage (*Brassica rapa* L. ssp*. pekinensis*) is a widely cultivated and economically important vegetable crop with typical leaf curvature. The TCP (Teosinte branched1, Cycloidea, Proliferating cell factor) family proteins are plant-specific transcription factors (TFs) and play important roles in many plant biological processes, especially in the regulation of leaf curvature. In this study, 39 genes encoding TCP TFs are detected on the whole genome of *B. rapa.* Based on the phylogenetic analysis of TCPs between *Arabidopsis thaliana* and *Brassica rapa*, TCP genes of Chinese cabbage are named from *BrTCP1a* to *BrTCP24b*. Moreover, the chromosomal location; phylogenetic relationships among *B. rapa*, *A. thaliana*, and rice; gene structures and protein conserved sequence alignment; and conserved domains are analyzed. The expression profiles of *BrTCPs* are analyzed in different tissues. To understand the role of Chinese cabbage TCP members in regulating the curvature of leaves, the expression patterns of all *BrTCP* genes are detected at three development stages essential for leafy head formation. Our results provide information on the classification and details of *BrTCPs* and allow us to better understand the function of *TCPs* involved in leaf curvature of Chinese cabbage.

## 1. Introduction

The TCP family proteins are widespread and specific transcription factors (TFs) in plants; examples include teosinte branched1 (TB1) from *Zea mays*, cycloidea (CYC) from *Antirrhinum majus*, and proliferating cell factor (PCF) from *Oryza sativa* [1,2,3,4,5] Based on phylogenetic analysis, TCP proteins are divided into two classes: class I and class II. Class I (TCP-P) is related to PCF1 and PCF2 proteins in rice, whereas class II (TCP-C) to CIN (Cincinatta in *Antirrhinum*) and the CYC/TB1 subgroups [6]. The *TCP* family genes encode a conserved region containing 59 amino acid residues with a basic helix–loop–helix (bHLH) structure, which are involved in DNA binding, protein–protein interaction, and protein nuclear localization [3]. However, the DNA binding sites of the two TCP classes are partly overlapping. Class I TCPs bind the DNA sequence GGNCCCAC, whereas class II GTGGNCCC [6].

The TCP family TFs regulate plant growth and development and lateral branching [7,8], leaf morphogenesis [8,9], flower development [10,11], embryo growth [12,13], circadian rhythm [14], and hormonal pathways [15,16]. In *Arabidopsis*, class I and II *TCP* members are involved in leaf development. The class II CIN-type genes, *AtTCP2*, *AtTCP3*, *AtTCP4*, *AtTCP10*, and *AtTCP24*, are regulated by miR319a and lead to serrated and crinkled leaves [17]. The repression of class I *AtTCP11* in the C-terminal domain results in curling of rosette leaves in *Arabidopsis* [16]. *AtTCP15* and its closest homologous gene *AtTCP14* play a negative role in the elaboration of leaf shape [18]. Moreover, TCP TFs have attracted the attention of the scientific community because of their function in developmental processes and defense response against biotic and abiotic stresses. *TCP13*, *TCP14*, and *TCP19* were remarkably found to participate directly in pathogenesis [19]. The *tcp15* mutant improves disease resistance to *HpaNoco2* in *Arabidopsis*. Furthermore, compared with other TCPs, TCP8, TCP14, and TCP15 interact more strongly with SRFR1, an adaptor protein in cytoplasmic microsomal and nuclear protein complexes. By contrast, class I TCP proteins with a conserved cysteine residue at position 20 (Cys-20) are sensitive to redox conditions, which affect the DNA-binding activity of TCP. In a previous study, a class I TCP protein OsPCF2 induced by salt stress was shown to participate in salt stress by binding the promoter of *OsNHX1* to activate its expression [20]. *NHX1* encodes a kind of K^+^-Na^+^/H^+^ antiporter, and its overexpression enhances the tolerance of salt and drought stress in *Arabidopsis* and rice [21]. Moreover, the overexpression of *OsTCP19* improves the tolerance of *Arabidopsis* under drought stress by modulating the ABI4-mediated pathways [22]. Thus, based on the above findings, TCP proteins play a significant role in developmental processes and defense against biotic and abiotic stresses.

Chinese cabbage (*Brassica rapa* L. ssp*. pekinensis*) is a widely cultivated and economically important vegetable crop in Asia. Chinese cabbage originates in China, and it has now become increasingly popular in other countries [23]. The vegetative periods of Chinese cabbage are divided into four stages: germination, seedling, rosette, and heading. Leafy head formed at the heading period is the storage organ and commonly the eaten part of the cabbage. The yield and quality of cabbage are generally measured by the size and solidity of the head. Head formation is affected by the direction of leaf curvature, and more and more research on the heading mechanism of cabbage has been made. Previous studies speculated that head formation is caused by the asymmetrical distribution of auxin [24]. In another study, some genes of the *BrLAX*, *BrPIN*, and *BrPGP* families have been found to play important roles in the asymmetrical auxin distribution by affecting the polar auxin transport during leafy head development [25]. Furthermore, in accordance with the work model of leaf shape diversity regulated by adaxial–abaxial (Ad–Ab) polarity in *Arabidopsis* [26], six leaf Ad–Ab patterning candidate genes were selected between heading and non-heading Chinese cabbages, namely *BrARF3.1*, *BrARF4.1*, *BrKAN1*, *BrKAN2.1*, *BrRDR6*, and *BrHYL1.1*, which are linked to head formation [27]. Some genes are known to participate in regulating the shape of leaves in *Arabidopsis*; *BrpTCP4* was particularly reported to regulate the head shape of Chinese cabbage by miR319a [9].

As the whole genome of Chinese cabbages is sequenced [28], genome-wide analysis of *TCP* genes is performed for the first time in our study. In the present study, the TCP TF family in Chinese cabbage is comprehensively analyzed. We identify a total of 39 genes encoding TCP TFs on the whole genome of *Brassica rapa.* By associating the phylogenetic analysis of TCP domain proteins between *Arabidopsis thaliana* and *Brassica rapa*, *BrTCP1a* to *BrTCP24b* are named. Then, the chromosomal location; phylogenetic relationships among *B. rapa*, *A. thaliana*, and rice; gene structures and protein conserved sequence alignment; and conserved domains were analyzed. The expression profiles of *BrTCPs* are detected in different tissues. To determine the role of Chinese cabbage *TCP* members in regulating the curvature of leaves, the expression patterns of all *BrTCP* genes are detected at three essential development stages. The results of our study provide information on the classification and details of *BrTCPs* and lay the foundation for future studies on the leaf curvature mechanism of TCP proteins in Chinese cabbage.

## 2. Results

### 2.1. Identification of TCP TFs in Chinese Cabbage

A conserved TCP domain is usually found at the N terminal of TCP TFs. To obtain the TCP members of Chinese cabbage, the TCP domains (PF03634) of the *B. rapa* genome were searched on the Brassica Database (BRAD), and all TCP candidate sequences were examined using the domain analysis online tools: PfamScan (https://www.ebi.ac.uk/Tools/pfa/pfamscan/), InterPro (a protein sequence analysis and classification website, http://www.ebi.ac.uk/interpro/), and SMART (http://smart.embl-heidelberg.de/). As a result, 43 genes were found with a typical TCP domain, and 39 genes with no redundant sequences were screened. These genes were confirmed to contain the TCP domain with the ScanProsite tool. They were named *BrTCP1* to *BrTCP24* according to the *TCP* gene classifications of *A. thaliana* (Table S1). However, no homologous genes of *AtTCP11*, *AtTCP16*, and *AtTCP23* were found in Chinese cabbage. The main TCP genetic characteristics of *B. rapa* are summarized in Table 1, including the gene names, gene ID, chromosome location, full length of cDNA, exon numbers, protein length, molecular weights, and isoelectric points. For all *BrTCPs*, the protein length ranged from 176 aa to 538 aa, the molecular weights from 18,601 Da to 59,180.4 Da, and the isoelectric points from 5.419 to 10.8713 as shown in Table 1.

### 2.2. Chromosomal Location and Distribution of TCP Genes in Chinese Cabbage

**Figure 1:**
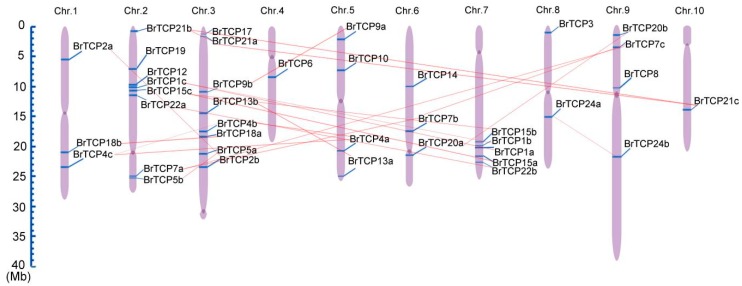
The chromosomal location of *TCP* genes from Chinese cabbage. The scale represents 40 Mb chromosomal distance. The chromosome numbers are labeled on the top of them. The duplicated gene are connected with red lines.

The physical chromosomal location of *TCP* genes was searched on BRAD (the *Brassica* database) by using BLASTN (http://brassicadb.org/brad/index.php), as shown in Figure 1. All 39 members of Chinese cabbage TCPs were unevenly distributed in all 10 *B. rapa* chromosomes, containing one to eight genes per chromosome. Four *TCP* genes were each found on chromosomes A5 and A9. The largest number (nine) of *BrTCPs* was located on chromosome A2 and A3, followed by five *BrTCP* genes in A7. There were two *BrTCPs* on A8. Only one gene was located in A4 and A10. Chromosome A1 and A6 each had seven *BrTCPs*. Duplication analysis showed that nearly 80% (31 out of 39) of *BrTCP* genes existed in two or three copies. However, we found that most paralogous genes were distributed on different chromosomes. Both *BrTCP15a* and *BrTCP15b* were located on the long arm of chromosome A7, and *BrTCP1a* and *BrTCP1b* genes were located between them, however *BrTCP1a* and *BrTCP1b* were tandem on A7, suggesting that duplicate events in Chinese cabbage were caused by both segment duplications and tandem. Eight (33%), three (13%), six (13%), one (4%), and six (13%) *AtTCPs* were found in Chr.1 to 5 of *A. thaliana*, respectively, and three (7%), eight (20%), eight (20%), one (3%), and four (10%) *BrTCPs* were found in Chr.1 to Chr.5, respectively. The remaining *BrTCPs* (38%) were found in Chr.6 to Chr.10 (Table S2 and Figure S1). This finding suggests that the duplicate events of the *BrTCP* family were caused by the expansion of chromosomes in Chinese cabbage.

### 2.3. Phylogeny and Gene Structure of the BrTCP Family

**Figure 2:**
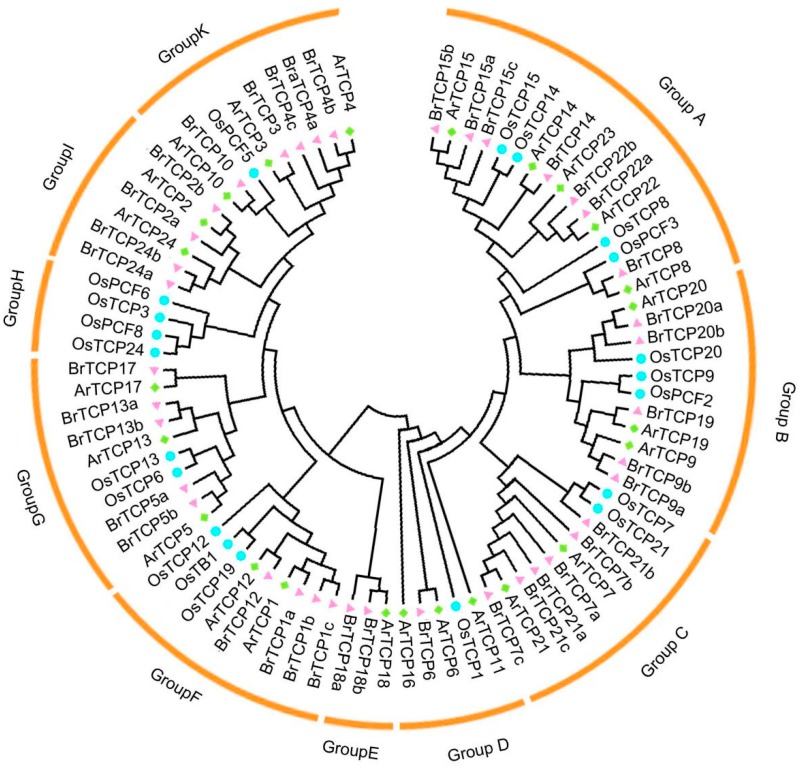
Phylogenetic relationships of TCP transcription factors among *Brassica rapa*, *Arabidopsis thaliana* and *Oryza. sativa*. The unroot phylogenetic tree was constructed by MEGA 6.0 (Tokyo Metropolitan University, Tokyo, Japan) using Neighbor-Joining method with 1000 bootstrap replicates. TCPs of different plants are indicated with different colors and shapes.

To better understand the phylogenetic relationships of *TCP* genes in Chinese cabbage, *Arabidopsis*, and rice, a neighbor-joining (NJ) phylogenetic tree was built based on multiple sequence alignment of 39 *B. rapa*, 24 *A. thaliana*, and 21 *Oryza sativa* genes with MEGA6 (Figure 2). All *TCP* genes were divided into seven subgroups based on their sequence features: group A to G. Groups A–D belonged to the class I subfamily, which is also called the PCF subfamily, and 13 *Arabidopsis* and 19 *Brassica rapa TCP* genes were found (Table 2). There were similar gene distribution in turnip, which has a close genetic relationship with Chinese cabbage. By contrast, groups E to G belonged to class II, which consists of two types: CIN and CYC/TB type. Furthermore, 29 CIN-type and 9 CYC/TB type members were found, representing 36.3% and 11.3% of all *TCP* members, respectively; however, 8 and 14 *AtTCPs* were found in CIN-type and CYC/TB type members of *Arabidopsis*, respectively. The largest number of *TCP* members belonged to the PCF type, which occupied 52.5%. Sixteen TCP proteins were found (20%) in group A, the largest subgroup, which also belonged to the PCF type. Meanwhile, the smallest subgroup was the CYC/TB type, which contained three BrTCP proteins (4%) but only one *Arabidopsis TCP* member. This finding suggested that the *TCP* family expanded before divergence of the lineages. The Chinese cabbage *TCP* genes were highly homologous to the *TCP* genes of *A. thaliana*. In addition, one *Arabidopsis TCP* gene may have at least one copy or even two or three copies in cabbage. However, *BrTCP11* and *BrTCP16* were not found in Chinese cabbage, which suggested that they not only underwent replication but also deletion of chromatin during evolution of Chinese cabbage.

**Figure 3:**
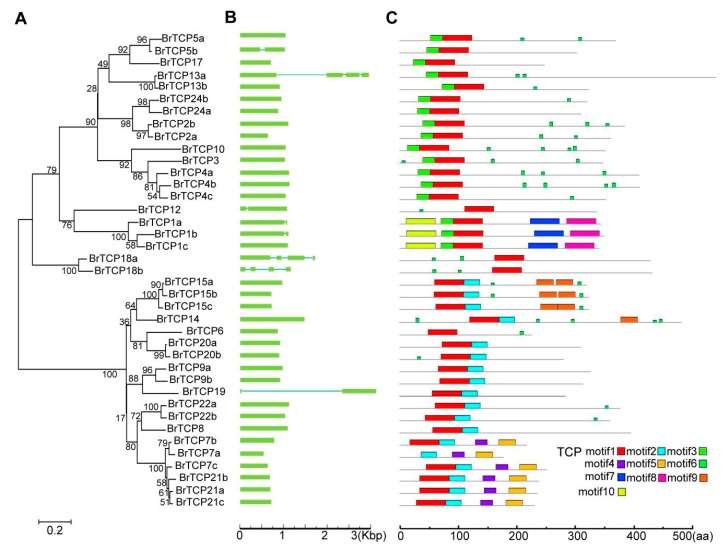
The Phylogenetic tree, gene structure and multiple motifs of TCP family in Chinese cabbage. (**A**) Phylogenetic tree was constructed based on the protein sequence of Chinese cabbage by MAGE6.0; (**B**) The gene structure of Chinese cabbage *TCPs*. Exon and intron were represented by green boxes and lines; (**C**) The multiple conserved motifs of Chinese cabbage TCP proteins. Motifs were identified by an online tool MEME and labeled with colored boxes. Motifs 1–3 belong to the TCP subfamily.

The structure of *BrTCP* genes was analyzed by alignment of cDNA and genomic DNA sequences, as shown in Figure 3B. Most Chinese cabbage *TCP* genes (31 out of 39) contained only one exon without an intron, and six *BrTCP* members contained two exons and one intron. In addition, *BrTCP13a*, *BrTCP18a*, and *BrTCP18b* contained four exons and three introns. Most *BrTCP* genes had similar exon/intron structures and distribution in the same phylogenetic subfamily, which were confirmed by the phylogenetic tree (Figure 3A).

### 2.4. Conserved Domains and Motif Analysis of Chinese Cabbage

The conserved motifs of Chinese cabbage TCP proteins were confirmed by InterPro and analyzed using the MEME program (The University of Queensland, Brisbane, Australian) [29]. Ten putative motifs were identified by MEME analysis, namely, motifs 1–10. Motifs 1–3 were all identified as the TCP domain; however, motifs 4–10 were unknown domains (Figure 3A and Figure S2). Nearly all BrTCPs contained motif 1, except BrTCP7a, which should be an essential motif in BrTCPs. Motif 3 existed in almost every class I BrTCP, except BrTCP6, whereas most class II BrTCPs contained motif 2, except BrTCP12, BrTCP18a, and BrTCP18b, which belonged to the CYC/TB type. Among them, an R domain was present, which was predicted to be a coiled coil domain mediating protein–protein interactions (Figure S3). As a result of our identifications, BrTCPs of the same subfamily contained similar motif compositions, but they differed from different subfamilies, which suggested that different subfamilies have complementary functions, but the same subfamily exhibits redundancy.

**Figure 4:**
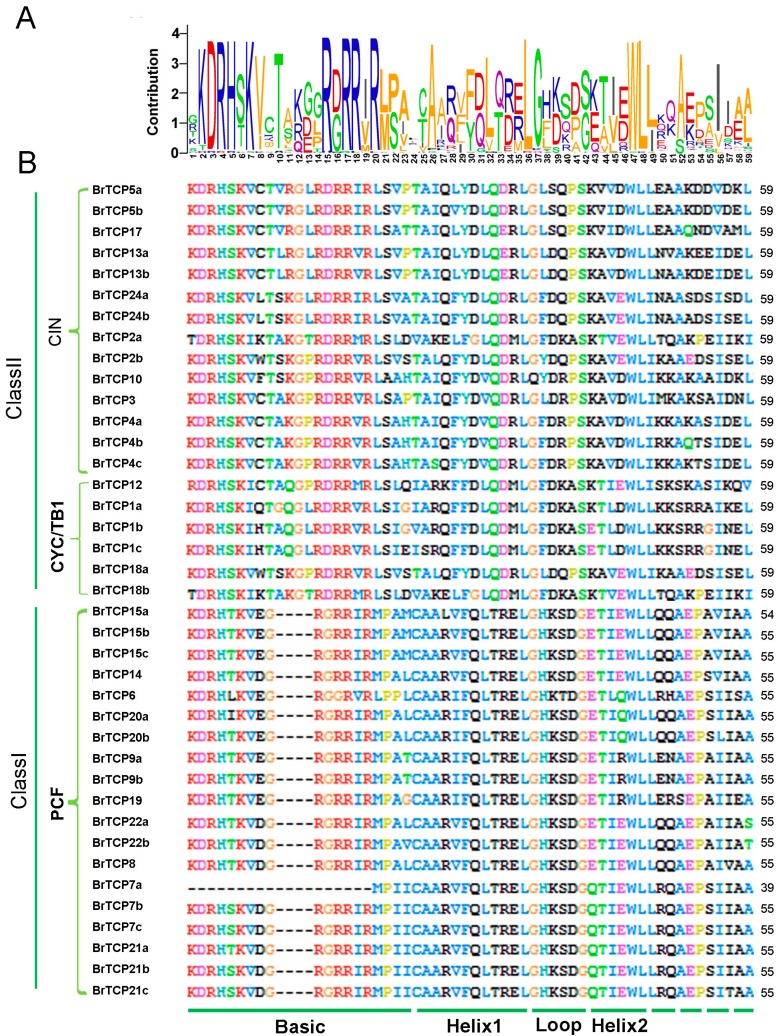
Multiple protein sequences alignment of TCP domain in Chinese cabbage. (**A**) Sequence LOGO of *TCPs* in Chinese cabbage. The LOGO representation created by Weblogo online software (Berkeley University, CA, USA) based on BrTCP protein sequences; (**B**) Multiple sequence alignment of Chinese cabbage TCP motifs by Clustal X2(University College Dublin, Dublin, Ireland).

The TCP domain was reported to participate in dimerization and DNA binding by a bHLH motif with usually 59 amino acid residues [3]. TCP domains were found in all BrTCPs and consisted of 39 to 59 amino acid residues with a bHLH structure (Figure 4). In the class I BrTCP subfamily, four amino acid deletions were found relative to that in the class II subfamily at the basic region. Sequences between the class I and II subfamilies were quite different in helix I, loop, and helix II, whereas a conservative tandem of W (tryptophan) and L (leucine) was found in helix II. These results revealed that different subfamilies have complementary functions, while the same subfamilies exhibit redundancy.

### 2.5. Expression Levels of TCP Genes in Chinese Cabbage

**Figure 5:**
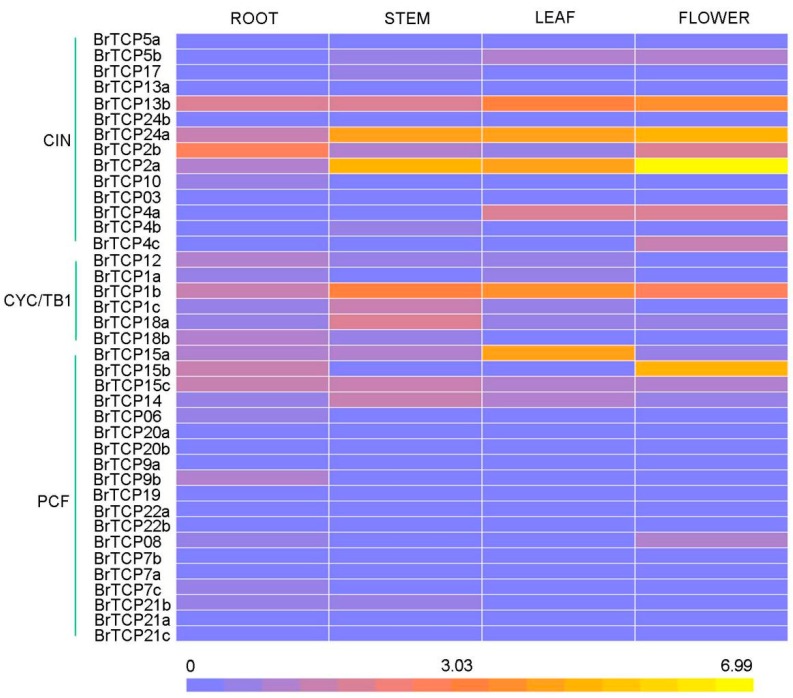
Expression patterns of Chinese cabbage TCP genes in different tissues represented by heat map. The expression level of *BrTCP* genes in root, stem, leaf and flower were detected by quantitative real-time PCR.

*TCPs* from *A. thaliana* were reported to be involved in the regulation of leaf morphology, including the shape and curvature of leaves [16,25]. To explore the potential function of *TCP* genes in Chinese cabbage, the expression levels of all 39 *BrTCP* genes were detected in root, stem, leaf, and flower tissues of “Dongnong A160” cabbage by real time polymerase chain reaction (RT-PCR). Total mRNAs were isolated at the reproductive stage, and all data were normalized with *Actin* as an internal control (Figure 5). The expression levels of different subfamily *BrTCP* genes varied significantly in these four tissues. *BrTCP1b*, *BrTCP2a*, *BrTCP5b*, *BrTCP15a*, *BrTCP15c*, and *BrTCP24a* were highly expressed in all four tissues. However, the expression levels of *BrTCP15b* were higher in flower than in other tissues, which was the same as *BrTCP4c.* Moreover, *BrTCP4a* was highly expressed in leaf and flower tissues. Most PCF subfamily genes showed low expression levels. The differential expression levels of *BrTCPs* in specific tissues indicated that these genes played multiple regulatory roles at different development stages.

### 2.6. Associated Relationship between the Expression of BrTCPs and Heading of Chinese Cabbage

**Figure 6:**
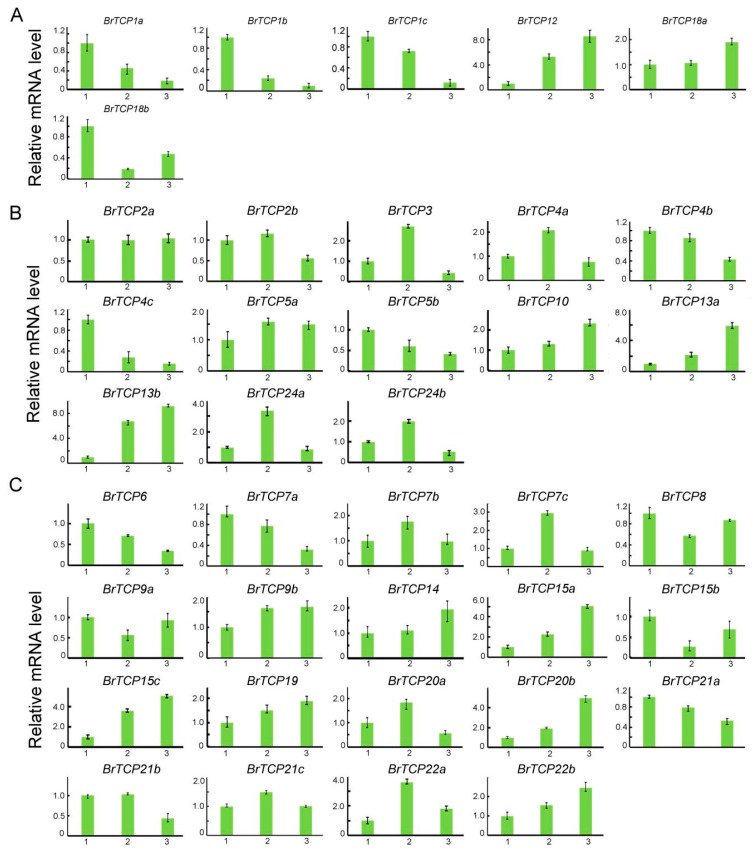
The expression level of *BrTCP* genes in Chinese cabbage selfing line A160 at the stage of seedings, rosette and headings: (**A**) the expression level of CyC/TB1 type *BrTCP* genes A160 at the stage of seedlings, rosette and headings were detected by quantitative real-time PCR; (**B**) the expression level of CIN type *BrTCP* genes A160 at the stage of seedlings, rosette and headings; and (**C**) the expression level of PCF type *BrTCP* genes A160 at the stage of seedlings, rosette and headings. Numbers 1,2 and 3 represent the stages of seedling, rosette and heading, respectively. The represent results performed in triplicate. Error bars represent ± SE

During the development of Chinese cabbage, heading is important for the formation of leafy head and cabbage yield. The TCP family is known to be involved in regulating the development of leaf curvature [16,25]. To gain insights on the relationship between heading and the TCP family in Chinese cabbage, the expression levels of *BrTCPs* were detected at the seeding, rosette, and heading stages of Chinese cabbage “Dongnong” A160 by RT-PCR. All *BrTCPs* were divided into three groups according to the subfamily, CYC/TB1, CIN, and PCF. For the CYC/TB1 type, six *BrTCPs* were detected, and four genes were downregulated at the rosette and heading stages compared with the seedling stage. Especially, *TCP1a*, -*b*, and -*c* were decreased by tenfold at the heading stage. However, *BrTCP12* was upregulated by 5.3- and 8.5-fold at the rosette and heading stages, respectively (Figure 6A). In the CIN-type TCP family, *BrTCP3*, *BrTCP4a*, and *BrTCP24a* were significantly upregulated at the rosette leaf stage compared to the other two stages. At the rosette stage, *BrTCP13a* and *BrTCP13b* were upregulated by 2.18- and 6.7-fold, respectively, and were even higher at the heading stage (5.8- and 9.1-fold). However, the expression of *BrTCP4c* dramatically decreased to 6.5-fold at the heading stage (Figure 6B). For the PCF type, the expression levels of *BrTCP7a* decreased gradually from seeding to heading. At the same time, *BrTCP7b* and *BrTCP7c* were enhanced by up to 2.9-fold at the rosette stage. Furthermore, *BrTCP15a* and *BrTCP15c* increased by 2.3- and 3.6-fold at the rosette stage, respectively, and both increased by fivefold at the heading stage. However, *BrTCP15b* decreased by 3.7-fold at the rosette stage, while *BrTCP22a* was upregulated by 3.6-fold. In addition, *BrTCP20b* was increased by fivefold at the heading stage (Figure 6C).

**Figure 7:**
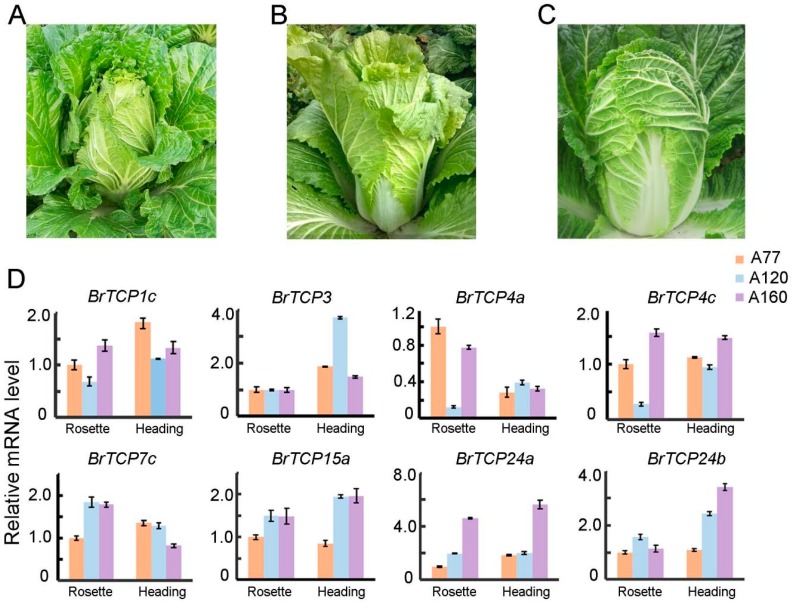
The expression level of *BrTCP* genes in leaves of different heading types of Chinese cabbages: (**A**) the inbred line of A120 Chinese cabbage at heading stage; (**B**) the inbred line of A77 Chinese cabbage at heading stage; (**C**) the inbred line of A160 Chinese cabbage at heading stage; and (**D**) the expression level of *BrTCP1c*, *BrTCP3*, *BrTCP4a*, *BrTCP4c*, *BrTCP7c*, *BrTCP15a*, *BrTCP24a*, and *BrTCP24b* in the inbred lines of A120, A77, and A160 at rosette and heading stages. The represent results performed in triplicate. Error bars represent ± SE

Several *A. thaliana TCP* members were reported to be involved in regulating the curvature of leaves, such as *AtTCP2*, *AtTCP3*, *AtTCP4*, *AtTCP10*, *AtTCP24*, and *AtTCP15* [7,8,13,16,18,26]. In terms of phylogenetic analysis and the differential expression of *BrTCPs* between the rosette and heading stage, eight *BrTCP* genes were selected to measure the expression levels among different heading-type cabbages, namely, *BrTCP1c* homologous to *AtTCP1*, *BrTCP3* homologous to *AtTCP3*, *BrTCP4a* and *-4c* homologous to *AtTCP4*, *BrTCP7c* homologous to *AtTCP7*, *BrTCP15a* homologous to *AtTCP15*, and *BrTCP24a* and *-24b* homologous to *AtTCP24.* Leafy heads of Chinese cabbage were generally divided into three types: the fold heading type (A77 inbred line) with the involute leaves less than the axle wire (Figure 7A), overlapping heading type (A160 inbred line) with the involute leaves exceeding the axle wire (Figure 7C), and straight heading type (A120 inbred line) with revolute leaves (Figure 7B). The expression levels of *BrTCP1c*, *BrTCP4a*, and *BrTCP4c* were lower in A120 than in A77 and A160, among which *BrTCP4a* and *BrTCP4c* were downregulated by 8.3- and 3.7-fold, respectively. However, *BrTCP3* in A120 was upregulated by 3.7-fold at the heading stage. *BrTCP24a* in A160 was upregulated by 4.6- and 5.7-fold at the rosette and heading stages, respectively, and the expression of *BrTCP24a* in A160 was only upregulated by 3.4-fold at the heading stage (Figure 7D). No obvious changes were found in *BrTCP7c* and *BrTCP15a.* These results revealed that *BrTCPs* may be involved in different pathways and development stages to regulate the curvature of leaves.

## 3. Discussion

The TCP TFs are a kind of plant-specific TF family, which participate in multiple functions in plant growth and development. A number of TCP TFs have been identified with genome-wide analysis, for example, *Arabidopsis*, rice, and other plants and even turnips *(B. rapa* ssp.), which had close relationship with Chinese cabbage [30,31,32]. However, no information on Chinese cabbage TCP TFs is available. In our study, 39 *BrTCP* members were identified from the Chinese cabbage genome, and the number of *TCPs* in Chinese cabbage was more than that of *Arabidopsis*, while in *Arabidopsis* 24 *TCPs* were found. Both *Arabidopsis* and Chinese cabbage belong to the *Brassicaceae* family, and Chinese cabbage is also a subspecies of *B. rapa*, which has undergone polyploidization, leading to additional whole genome triplication [27]. The *BrTCP* gene family was expanded by 1.625-fold, and two or three copies were homologous to one *AtTCP* protein. Eight *BrTCPs* contained a single duplication, *BrTCPs* contained a single duplication, nine sister pairs and four of three duplications. However, three *BrTCPs* were missing in the Chinese cabbage: *BrTCP11*, *BrTCP16* and *BrTCP23* (Table S1). All these *BrTCPs* were segmentally duplicated and unevenly distributed on the genome, which played a role in genomic rearrangement and diversification. In addition, turnip also had 39 TCPs, but there were some differences in duplicated copies between Chinese cabbage and turnip. *TCP2*, *TCP5*, and *TCP22* were single copy in turnip, however they were two duplicated copies in cabbage. Conversely, there was only one *BrTCP17* but two *BrrTCP17*. *TCP23* was missing in cabbage, while turnip had a *TCP23*. There were three *BrrTCP20* and two *BrTCP20*. These different duplicated copies indicate that TCP may play distinguishing role in biological process between Chinese cabbage and turnip even their close correlation between relatives.

TCP family TFs were divided into three subfamilies according to their conservative domains, including PCF, CIN, and CYC/TB1 [3]. The TCP family in Chinese cabbage also consisted of these three parts: PCF with 1.5-fold expansion compared to *Arabidopsis* and containing 19 members but 20 members in turnips, CIN with 1.8-fold expansion and 14 members but 13 members in turnips, and CYC/TB1 with twofold expansion and six members, the same as in turnips (Table 2). Almost all the *BrTCPs* contained the conservative motif 1, except for *BrTCP7a*, which was obviously shorter than other proteins. Besides motif 1, the PCF subfamily usually contains motif 2 in front of motif 1 in proteins. Motif 3 was found in both the CIN and CYC/TB1 subfamilies. Furthermore, *BrTCP12*, *BrTCP18a*, and *BrTCP18b* of the CYC/TB1 subfamily contained an R domain (Figure 3C and Figure 3). In addition, motifs 1–3 were specific to the TCP domain, which was formed by a bHLH motif of 54–59 amino acid residues. These indicated that BrTCPs may play different roles in growth and development, although they were in the same TCP subfamily. However, most BrTCP proteins of the same subfamily shared not only relatively similar conservative domain but also similar gene structures. Next, the expression pattern of each *BrTCP* subfamily was detected in root, stem, leaf, and flower tissues by qRT-PCR. Most *BrTCP* genes of the PCF subfamily were downregulated in these tissues, except for *BrTCP15a* and *BrTCP15b*. *BrTCP1c* of CYC/TB1 was apparently upregulated, and the majority of upregulated genes were found in the CIN subfamily, which indicate *BrTCPs* of CIN subfamily play a vital role in head formation of Chinese cabbage. The expression pattern and distribution of conserved domain were related to their molecular functions. These results indicated that single or pairs of genes of the same subfamily may play similar or complementary roles in biological processes.

TCP family TFs are involved in the regulation of leaf curvature in many plants. *AtTCP2*, *AtTCP3*, *AtTCP4*, *AtTCP10*, and *AtTCP24* of the CIN subfamily were the targeted genes of miR319, and they modulate leaf development in *Arabidopsis* [12,13,18,29]. In terms of the close phylogenetic relationship between *Arabidopsis* and Chinese cabbage, *BrTCP* genes might play a role in leaf morphogenesis. To explore the correlation between *BrTCP* genes and leaf curvature, we monitored the expression levels of 39 *BrTCP* genes at the seedling, rosette, and heading stages in the inbred line A160, which is a typical “overlapping” cabbage. For CYC/TB1 subfamily, the expression of *BrTCP1a*, *BrTCP1b*, *BrTCP1c*, *and BrTCP18b* were remarkable downregulated at the rosette and heading than at the seedling stage, but *BrTCP12* and *BrTCP18a* got the maximum on the heading stage. Most of the CIN subfamily members were the taget genes of miR319, such as *BrTCP2*, *BrTCP3*, *BrTCP4*, *BrTCP10*, and *BrTCP24. BrTCP2*, *BrTCP3*, *BrTCP4a*, *BrTCP5a*, *BrTCP24a*, and *BrTCP24b* upregulated to the peak on the rosette stage. However, the highest expression of *BrTCP10* was observed at heading stage. *BrTCPs* have discrepant functions in heading formation even in the same subfamily, which indicated that these genes were involved in heading formation caused by the remarkable differential expressions *BrTCP15a* and *BrTCP15c* increased gradually during Chinese cabbage development. TCP14 and TCP15 act as repressors of cell proliferation in the developing leaf in *Arabidopsis* [18], and *BrTCP15a* and *BrTCP15c* increased gradually during Chinese cabbage development with no obvious expression change of *BrTCP14*, suggested that *BrTCP15* played a dominant role in Chinese cabbage. To further analyze the relationship between the expression levels and different shape heads, eight *BrTCPs* were selected for further determination in the inbred lines A77, A120, and A160. It was reported that *TCP1* played a positive role in BR biosynthesis pathway by regulating the expression of DWF4 [15] and involved in longitudinal elongation of leaves in *Arabidopsis* [33]. We showed that the expression level of *BrTCP1* were higher in heading cabbage, A77 and A160, than straight cabbage in both rosette and heading stages, which indicated that BR may played a role in leaf curvature to regulate heading formation. Further studies are required. The expression of *BrTCP3* was upregulated only in A120 at the heading stage. Whereas *BrTCP4a* in A120 was downregulated at the rosette stage, and the expression of *BrTCP4c* was highest in overlapping heading type (A160), secondly folding type (A77) and lowest in straight type (A120), which were agreed with the reported gene *BrpTCP4* in Chinese cabbage. *BrpTCP4* showed low expression level in cylindrical head shape and high in round head shape, which was reported to regulate the size and shape of leafy heads in Chinese cabbage [9]. The double mutant of *tcp2 tcp4* showed enlarged flat leaves, however the *tcp2 tcp3 tcp4 tcp10* mutant had strongly crinkled leaves in *Arabidopsis* [34]*.* The expression pattern of *BrTCP3* and *BrTCP4* seemed to be opposite among different heading types, which revealed the different *BrTCPs* played diverse role in leaf curvature. Moreover, the expressions of *TCP24* were elevated in the *p35S*:*mTCP24* transgenic *Arabidopsis*, which showed rosette leaves slightly downward [35]. *BrTCP24a* in overlapping heading (A160) was highest at both the rosette and heading stages; the expression level of *BrTCP24b* in A160 was highest compare to others on the heading stage, that suggested that the function of *BrTCP24* were similar with *AtTCP24* in leaf curvature. Our results revealed that *BrTCP* genes played either a synergistic, complementary, or opposite role in the formation of leaf curvature. Therefore, further studies are needed to determine their specific functions.

## 4. Materials and Methods

### 4.1. Identification and Analysis of TCP Family Members in Chinese Cabbage

To identify the *TCP* members in Chinese cabbage, we searched for a conserved TCP domain (PF03634) based on the Hidden Markov Model on BRAD (http://brassicadb.org/brad/) and verified the *BrTCP* members on the BLAST(Basic Local Alignment Search Tool) program of the NCBI (National Center for Biotechnology Information) database (https://blast.ncbi.nlm.nih.gov/Blast.cgi). Full-length nucleotide and protein sequences of the TCP family in Chinese cabbage were also searched and obtained from BRAD (http://brassicadb.org/brad/). The TCP family sequences of *A. thaliana* and *O. sativa* were downloaded from TAIR (http://www.arabidopsis.org/) and the *O. sativa* Genome Annotation Project (http://rice.plantbiology.msu.edu/) and were adjusted with the plant TFDB (Plant Transcription Factor Databas)database (http://planttfdb.cbi.edu.cn/) [36].

### 4.2. Chromosomal Location and Gene Structure

The chromosomal location and gene structures of BrTCP genes were acquired from BRAD and were assessed and enhanced using MapInspect (Mike Lischke, Berlin, Germany) and Photoshop software CS (San Jose, CA, USA).

### 4.3. Phylogenetic Analysis and Analysis of Conserved Domains and Motif

The protein sequences of identified BrTCPs and *A. thaliana* and *O. sativa* TCP*s*were downloaded and compared to generate the phylogenetic tree. Multiple TCP sequence alignments were performed using Clustal X2.0 (University College Dublin, Dublin, Ireland) [37], and MEGA6 (Tokyo Metropolitan University, Tokyo, Japan) was used to construct the phylogenetic trees with the NJ (neighbor joining) method [38] by using 1000 replicates.

To identify the conserved motif of BrTCPs, all protein sequences were analyzed using the online tool MEME with the following settings: repetition number, any; maximum motif number, 5; motif width, between 6 and 50; and minimum motif width, 6. In addition, InterPro (http://www.ebi.ac.uk/interpro/scan.html) and Pfam (http://pfam.xfam.org/search#tabview=tab1) were used for further confirmation and annotation [29].

### 4.4. Plant Materials

“A160” were sown in the greenhouse until the cotyledon were unfolding, and then seedlings were vernalized for 20 days at 4 °C. The tissues of lateral root, stem, rosette leaf and flower were harvested when cabbages were flowering at 28 °C under a 16 h light/8 h dark cycle.

Seeds of selfing lines A77-1-1, A120-1, and A160 (fold heading, straight cabbage, and overlapping heading cabbage, respectively) were sown in the field on 20 July. A77 inbred line, A160 inbred line and A120 inbred line were fold heading type with the involute leaves less than the axle wire, overlapping heading type with the involute leaves exceeding the axle wire, and straight heading type with revolute leaves, respectively. The fifth leaf were harvested at five leaf stage for seedling leaves, and rosette leaves were obtained at 10 leaf stage. The green lobus cardiacus were selected when the leaves incurved to form headings. All plant materials were for RNA isolation. 

### 4.5. Total RNA Isolation and qRT-PCR Analysis

Total RNA of each tissue was extracted with the EasyPure Plant RNA Kit (TransGen, Beijing, China), and 2 mg of total RNA was reversed transcribed with TransScript One-Step gDNA Removal and cDNA Synthesis SuperMix (TransGen, Beijing, China). For RT-PCR, 15 mL total volume with 7.5 mL of SYBR Green Real-Time PCR Master Mix (TOYOBO, Osaka, Japan), 0.25 mL of specific primers, and 5 mL of cDNA samples with 30–50-fold dilution were used. For each RT-PCR, 15 mL of total volume with gene-specific primers was used. The amplification was performed on a Bio-Rad (Berkeley, CA, USA) IQ5 Multi-Color Real-Time PCR Detection System. The reactions of tubulin were considered as internal references. All primers were listed in Supplementary Table S3. 

## Figures and Tables

**Table 1:** TCP family transcription factor genes in Chinese cabbage.

Gene Name	Gene ID	Chromosome Location	CDS Length (bp)	Exon	Protein length (aa)	*M*_W_ (Da)	PI
*BrTCP1a*	Bra004212	A07:20728546–20729668	1026	2	341	38,786.3	6.5294
*BrTCP1b*	Bra004097	A07:20111066–20112206	1044	2	347	39,380	6.1198
*BrTCP1c*	Bra034010	A02:10223409–10224449	1041	1	346	38,511.8	5.477
*BrTCP2a*	Bra013304	A01:4882809–4883528	720	1	239	39,219.7	8.3134
*BrTCP2b*	Bra012600	A03:23315387–23316535	1149	1	382	42,262.9	7.6883
*BrTCP3*	Bra030952	A08:1031146–1032183	1038	1	345	37,595.3	7.2706
*BrTCP4a*	Bra027284	A05:20829037–20830257	1221	1	406	44,035.4	7.5321
*BrTCP4b*	Bra001579	A03:17191721–17192944	1224	1	407	44,472.9	8.0355
*BrTCP4c*	Bra021586	A01:23505568–23506620	1053	1	350	38,203.2	7.3461
*BrTCP5a*	Bra012990	A03:21199353–21200453	1101	1	366	40,698.2	6.6787
*BrTCP5b*	Bra029344	A02:25211660–25212733	906	2	301	33,796.16	6.5
*BrTCP6*	Bra025528	A04:8476440–8477114	675	1	224	24,724.6	7.816
*BrTCP7a*	Bra029366	A02:25051573–25052103	531	1	176	18,601	8.2872
*BrTCP7b*	Bra009667	A06:17073181–17073933	753	1	250	26,968.1	10.3355
*BrTCP7c*	Bra026506	A09:3299278–3299928	651	1	216	23,116.8	9.867
*BrTCP8*	Bra027886	A09:10035227–10036411	1185	1	394	41,385.3	6.5677
*BrTCP9a*	Bra004929	A05:2539838–2540812	975	1	324	34,510.1	10.1796
*BrTCP9b*	Bra000395	A03:10937732–10938667	936	1	311	33,174.9	10.2854
*BrTCP10*	Bra018280	A05:7148195–7149244	1050	1	349	38,727.4	6.8541
*BrTCP12*	Bra038350	A02:10071368–10072479	1008	1	335	38,247.8	8.7635
*BrTCP13a*	Bra039158	A05:25073263–25074192	930	1	309	35,797.8	7.4173
*BrTCP13b*	Bra001032	A03:14476673–14479645	1617	4	538	59,180.4	7.2645
*BrTCP14*	Bra018126	A06:10447686..10449086	1400	1	466	51,551	7.2085
*BrTCP15a*	Bra004407	A07:21777258–21778223	966	1	321	34,066.1	7.4651
*BrTCP15b*	Bra003986	A07:19409073–19409810	738	1	245	33,798.6	7.7239
*BrTCP15c*	Bra007875	A02:10484033–10484773	741	1	246	33,870.94	7.12
*BrTCP17*	Bra005967	A03:1433460–1434200	741	1	246	27,367.4	7.0159
*BrTCP18a*	Bra001710	A03:18008822–18010603	1278	4	425	48,457.2	8.3237
*BrTCP18b*	Bra037579	A01:21234671–21236261	1284	4	428	48,910.3	7.1925
*BrTCP19*	Bra022568	A02:7842256–7845540	894	2	297	30,182.5	5.419
*BrTCP20a*	Bra025244	A06:21530080–21531003	924	1	307	32,446.8	7.954
*BrTCP20b*	Bra039096	A09:1525195–1526031	837	1	278	29,671.6	6.5716
*BrTCP21a*	Bra005984	A03:1498581–1499285	705	1	234	24,263.2	10.8713
*BrTCP21b*	Bra028662	A02:1265168–1265857	690	1	229	23,957.7	10.2007
*BrTCP21c*	Bra009338	A10:14235005–14235715	711	1	236	24,733.4	9.2658
*BrTCP22a*	Bra008001	A02:11473562–11474686	1125	1	374	39,098.2	8.9956
*BrTCP22b*	Bra016090	A07:22761310–22762380	1071	1	356	37,206.3	7.4347
*BrTCP24a*	Bra010789	A08:15682126–15683052	927	1	308	34,410.5	7.1472
*BrTCP24b*	Bra032365	A09:22194917–22195873	957	1	318	35,327.5	8.1305

**Table 2:** *TCP* family genes in *Arabidopsis thaliana*, Chinese cabbage and turnip.

	PCF	CIN	CYC/TB1	Total
*Arabidopsis thaliana*	13	8	3	24
*Brassica rapa* (Chinese cabbage)	19	14	6	39
*Brassica rapa* (turnip)	20	13	6	39

## Supplementary Materials

Supplementary materials can be found at http://www.mdpi.com/1422-0067/19/3/847/s1.
