# Supplementary material for: Genome-Wide Identification and Analysis of TCP Transcription Factors Involved in the Formation of Leafy Head in Chinese Cabbage
Source: Int J Mol Sci. 2018 Mar 14;19(3):847. doi: 10.3390/ijms19030847 (PMC5877708; doi:10.3390/ijms19030847)
Supplement: Supplementary file 1 [file ijms-19-00847-s001.pdf]

**Supplemetary Table1 Comparation of TCP Homologue Gene between *Brassica rapa* and *Arabidopsis thaliana.***

| Gene Name | Homologue Gene in Ar. | Identity (%) | Gene Name | Homologue Gene in Ar. | Identity (%) |
|---|---|---|---|---|---|
| *BrTCP1a* | | 71 | *BrTCP13a* | *AtTCP13* | 75 |
| *BrTCP1b* | *AtTCP1* | 70 | *BrTCP13b* | | 75 |
| *BrTCP1c* | | 70 | *BrTCP14* | *AtTCP14* | 76 |
| *BrTCP2a* | *AtTCP2* | 80 | *BrTCP15a* | | 84 |
| *BrTCP2b* | | 79 | *BrTCP15b* | *AtTCP15* | 86 |
| *BrTCP3* | *AtTCP3* | 60 | *BrTCP15c* | | 80 |
| *BrTCP4a* | | 76 | *BrTCP17* | *AtTCP17* | 67 |
| *BrTCP4b* | *AtTCP4* | 69 | *BrTCP18a* | *AtTCP18* | 70 |
| *BrTCP4c* | | 66 | *BrTCP18b* | | 68 |
| *BrTCP5a* | *AtTCP5* | 82 | *BrTCP19* | *AtTCP19* | 68 |
| *BrTCP5b* | | 73 | *BrTCP20a* | *AtTCP20* | 79 |
| *BrTCP6* | *AtTCP6* | 59 | *BrTCP20b* | | 65 |
| *BrTCP7a* | | 70 | *BrTCP21a* | | 80 |
| *BrTCP7b* | *AtTCP7* | 70 | *BrTCP21b* | *AtTCP21* | 83 |
| *BrTCP7c* | | 70 | *BrTCP21c* | | 80 |
| *BrTCP8* | *AtTCP8* | 75 | *BrTCP22a* | *AtTCP22* | 78 |
| *BrTCP9a* | *AtTCP9* | 69 | *BrTCP22b* | | 72 |
| *BrTCP9b* | | 67 | *BrTCP24a* | *AtTCP24* | 73 |
| *BrTCP10* | *AtTCP10* | 77 | *BrTCP24b* | | 67 |
| *BrTCP12* | *AtTCP12* | 67 | | | |

Ar. equals to *Arabidopsis thaliana.*

**Supplemetary Table 2 The locations of *AtTCPs* and *BrTCPs* on the Chromosome.**

| | Chr.1 | Chr.2 | Chr.3 | Chr.4 | Chr.5 | Chr.6 | Chr.7 | Chr.8 | Chr.9 | Chr.10 | Total |
|---|---|---|---|---|---|---|---|---|---|---|---|
| ***Arabidopsis thaliana*** | 8 | 3 | 6 | 1 | 6 | - | - | - | - | - | 24 |
| ***Brassica rapa*** | 3 | 8 | 8 | 1 | 4 | 3 | 5 | 2 | 4 | 1 | 39 |

**Supplemetary Table 3 Primers of qPCR.**

| Primer Name | Sequence (5'-3') | Primer Name | Sequence (5'-3') |
|---|---|---|---|
| *BrTCP1aF* | GGCATAGCAAGATTCAGACGG | *BrTCP1aR* | CGAAGAACTGGCGAGCAATT |
| *BrTCP1bF* | GCAAGATTCACACGGCACAA | *BrTCP1bR* | GAGATCGAAGAATTGGCGAGAA |
| *BrTCP1cF* | TTTACCTTTCTTCGCTGCCG | *BrTCP1cR* | TTTGACCCGGAGATGCTGTT |
| *BrTCP2aF* | GTGCTCACCCTTTCTCCGAC | *BrTCP2aR* | ATCCTGCCGGAGAAATCAGA |
| *BrTCP2bF* | TCCGATGAACGATGACGACA | *BrTCP2bR* | GACAACGAGTTAGCCGCAGC |
| *BrTCP3F* | GCGAGATCGTGGAGGTTGAA | *BrTCP3R* | TGGCCGTACAGACTTTGCTG |
| *BrTCP4aF* | GTTCTACGGGCAGAGCAATCC | *BrTCP4aR* | GCCACCAGCCTCTGAATTGA |
| *BrTCP4bF* | TTCTTCTTCAATGAGACGCCG | *BrTCP4bR* | TGACCTCCTTGCACCTCGAC |
| *BrTCP4cF* | GACTGGTGGCTTGTAACGGC | *BrTCP4cR* | TCGTCGGAGAAGGAGCAAAG |
| *BrTCP5aF* | TCATCGAGGCAATGGACCTC | *BrTCP5aR* | GTGTCTGTCTTTGCCACCGA |
| *BrTCP5bF* | TCCAGGTACCAATTTAGGTTTCTT | *BrTCP5bR* | CAAAACTCTCTCGCGGTGTTG |
| *BrTCP6F* | GTTGCCTCCTCTGTGTGCTG | *BrTCP6R* | CACTGGAGAGTCTCGCCGTC |
| *BrTCP7aF* | CGTCAAGCTGAGCCTTCCAT | *BrTCP7aR* | AGTGCATGTTGAGGTGGTGG |
| *BrTCP7bF* | ACGGAAGAGGGAGGAGGATC | *BrTCP7bR* | ATTTGTGGCCGAGCTCTCTC |
| *BrTCP7cF* | GAGCCTTCAATCATAGCCGC | *BrTCP7cR* | GTTTCCTCGGAGTGAGGCTG |
| *BrTCP8F* | GGTTCCTTCTTGGCTCAGCC | *BrTCP8R* | GGCAAGCACCCCAAGATTAG |
| *BrTCP9aF* | CGGCCATCGCTATGTCTGTT | *BrTCP9aR* | CCGAGTTATCTCCCACGTCAG |
| *BrTCP9bF* | CTCCACCGTCGTCGTCAATA | *BrTCP9bR* | TTCCTCTTCCTTCGACCTTGG |
| *BrTCP10F* | GTTATAGCGCCGGAGATGGA | *BrTCP10R* | GGTCTTTTCGTCCTGTGGCTC |
| *BrTCP12F* | CAACAACAGGAATGGTGATACCA | *BrTCP12R* | TTGAAATAGGGTTTCTCTCCTCATCTA |
| *BrTCP13aF* | TCGTCGTCGTGGATGAAATC | *BrTCP13aR* | TTTGCTGTGACGGTCTTTGC |
| *BrTCP13bF* | GGAGACGGCGGACCTCTT | *BrTCP13bR* | AGGAGAAGCCATGTAGAGAAGTAACA |
| *BrTCP14F* | TCTCCCTCCACCAACACCA | *BrTCP14R* | GCAAGTCTTTCTGCGGTTGTG |
| *BrTCP15aF* | ACGGTGAAACCATCGAGTGG | *BrTCP15aR* | TAGCCGGAATGGTTCCAGTC |
| *BrTCP15bF* | ATAAATCCGACGGCGAGACC | *BrTCP15bR* | TGGTTCCAGTCCCTGTAGCG |
| *BrTCP15cF* | TGGAACCATTCCGGCTAACT | *BrTCP15cR* | CGAAGATGAGCAGCGGAGAG |
| *BrTCP17F* | GAATCGTGAGAGCCTCGAGAA | *BrTCP17R* | TCCTGTCTCGAAGACCACGAA |
| *BrTCP18aF* | GCTGCGAGGAGTCTCAAACC | *BrTCP18aR* | CTCTATCCTCGACTGTCCCCAT |
| *BrTCP18bF* | GCATGACCGAAAATAAGGTGG | *BrTCP18bR* | TCTCCTTCAAAATGGGCGTT |
| *BrTCP19F* | GAAGGCACGACTCAAACGCT | *BrTCP19R* | CCCTGAGCCACCCAAAAACT |
| *BrTCP20aF* | TTTTGCGTCCATTCTTGGTG | *BrTCP20aR* | CCAACATTCCCTTCCTGAGC |
| *BrTCP20bF* | GTAAGAAGCAGCTTGCCCCC | *BrTCP20bR* | TCCTGATTCTCCGACCGC |
| *BrTCP21aF* | ACGAGCAACGACGGATCAGT | *BrTCP21aR* | CACGGCGTTAAGAGTTCCTTTG |
| *BrTCP21bF* | AAGGACCTCTTAACGCCGTCA | *BrTCP21bR* | TGCGAATTCTCCTCCCTCTC |
| *BrTCP21cF* | CTAACGGCGCCATAGTCGAG | *BrTCP21cR* | GGTCCTTAGCCGGTGGTTTC |
| *BrTCP22aF* | AGAACTCTCACGACCGTCCG | *BrTCP22aR* | GAACCGATCTTCCCGGTGAT |
| *BrTCP22bF* | GGGAACTATTCCGGCGAACT | *BrTCP22bR* | TGAGCAAAGACGGTTGAGCC |

| | | | |
|---|---|---|---|
| *BrTCP24aF* | CAAGACCGTCTTGGCTTCGA | *BrTCP24aR* | CAACTCGGAGATGGAGTCGG |
| *BrTCP24bF* | GCGGCTTCTGACTCAATCTCC | *BrTCP24bR* | CGTGCTCGACTTAGACAGCG |
| *BrActin3F* | GTACAACCGGCATCGTGCTT | *BrActin3R* | CATGTGGAAGTGCATAACCCTC |

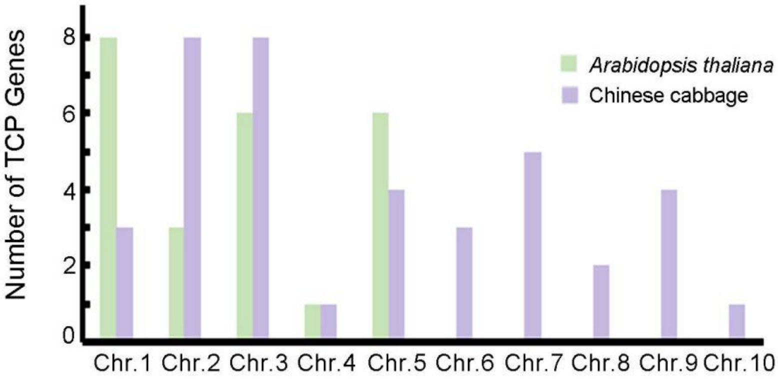


**Supplemetary Figure 1 The TCP number of *Arabidopsis thaliana* and *Brassica rapa* on each chromosome**

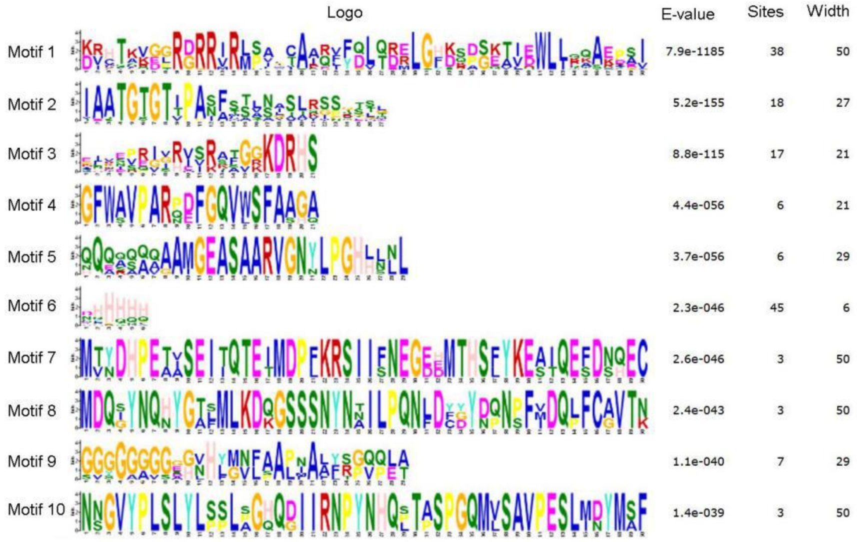


**Supplemetary Figure2 Sequence logos of motifs in *BrTCPs***

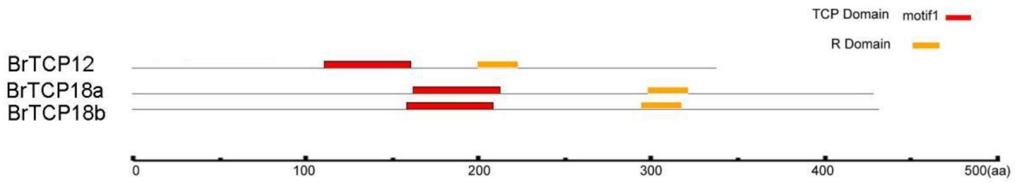


**Supplemetary Figure 3 The location of R Domain on *BrTCP12*，*BrTCP18a* and *BrTCP18a***
